# Consolidation of the Atlanta Medical College and the Southern Medical College

**Published:** 1898-06

**Authors:** 


### CONSOLIDATION OF THE ATLANTA MEDICAL COLLEGE AND THE SOUTHERN MEDICAL COLLEGE.

At this writing there is every indication that these well-known medical schools will be united. While the announcement of the consolidation has not been officially made and while the details of the union remain to be arranged, favorable negotiations are in progress which encourage the friends of medical education in general and of these institutions in particular to hope that amalgamation is in sight.

This is as it should be, and THE JOURNAL takes pleasure in congratulating those who have conceived and executed this wise and excellent movement.

It is likely that a new name will be selected for the consolidated college, and the new faculty will be composed of judicious selections from both the late faculties.

At present we are not able to make more than this preliminary announcement. By the next issue we hope to be able to report the final success of the plan and to present all the details of the consolidation.

---

THE business manager, Dr. Hutchins, attended the twenty-second annual meeting of the American Dermatological Association at Princeton, New Jersey, May 31st and June 1st. He also made a short visit to New York. Owing to his absence this issue of THE JOURNAL is mailed a few days late.

---

DR. FRANK S. BOURNS, of Atlanta, has been appointed Chief Surgeon on the staff of General Merritt and will accompany the latter's army of occupation to the Philippine Islands. The doctor lived in the Philippines a number of years ago.

